# Switchable radiative cooling and solar heating for sustainable thermal management

**DOI:** 10.1515/nanoph-2023-0627

**Published:** 2023-12-04

**Authors:** Myung Jin Yoo, Kyung Rok Pyun, Yeongju Jung, Minjae Lee, Jinwoo Lee, Seung Hwan Ko

**Affiliations:** Department of Mechanical Engineering, Seoul National University, 1 Gwanak-ro, Gwanak-gu, Seoul 08826, Republic of Korea; Electronic Devices Research Team, Hyundai Motor Group, 37 Cheoldobangmulgwan-ro, Ulwang-si, Gyeonggi-do 16082, Republic of Korea; Department of Mechanical, Robotic, and Energy Engineering, Dongguk University, 30 Pildong-ro-1-gil, Jung-gu, Seoul 04620, Republic of Korea; Institute of Advanced Machinery and Design (SNU-IAMD), Seoul National University, 1 Gwanak-ro, Gwanak-gu, Seoul 08826, Republic of Korea

**Keywords:** radiative cooling, solar heating, thermal management, switchable radiative cooling, sustainability

## Abstract

Radiative thermal management technologies that utilize thermal radiation from nano/microstructure for cooling and heating have gained significant attention in sustainable energy research. Passive radiative cooling and solar heating operate continuously, which may lead to additional heating or cooling energy consumption due to undesired cooling or heating during cold nighttime/winters or hot daytime/summers. To overcome the limitation, recent studies have focused on developing radiative thermal management technologies that can toggle radiative cooling on and off or possess switchable dual cooling and heating modes to realize sustainable and efficient thermal management. This review will explore the fundamental concepts of radiative thermal management and its switching mechanisms, utilizing novel systems composed of various materials and nano/microstructures. Additionally, we will delve into the potential future research directions in radiative thermal management technologies.

## 1 Introduction

Radiative thermal management, such as radiative cooling and solar heating, is considered energy-saving technology since it leverages thermal radiation between space and the Sun [1, 2]. Radiative cooling, an emerging technology, has garnered attention as a promising and sustainable cooling solution that harnesses the principles of Planck’s Law, which states that all physical bodies emit electromagnetic waves. Radiative cooling enables cooling without any energy consumption by harnessing the cold temperatures of outer space beyond atmosphere of the Earth (∼3 K), which acts as a heat sink. The atmosphere of the Earth has an atmospheric window in the 8–13 μm wavelength range where electromagnetic waves can pass through effectively, and objects at the Earth with ambient temperature (∼300 K) can efficiently absorb and emit radiation in this range. Solar energy, which accounts for 99.9 % of sustainable energy of the Earth, can also play a crucial role in zero-energy thermoregulation through efficient radiative solar heating [3].

**Figure 1:**
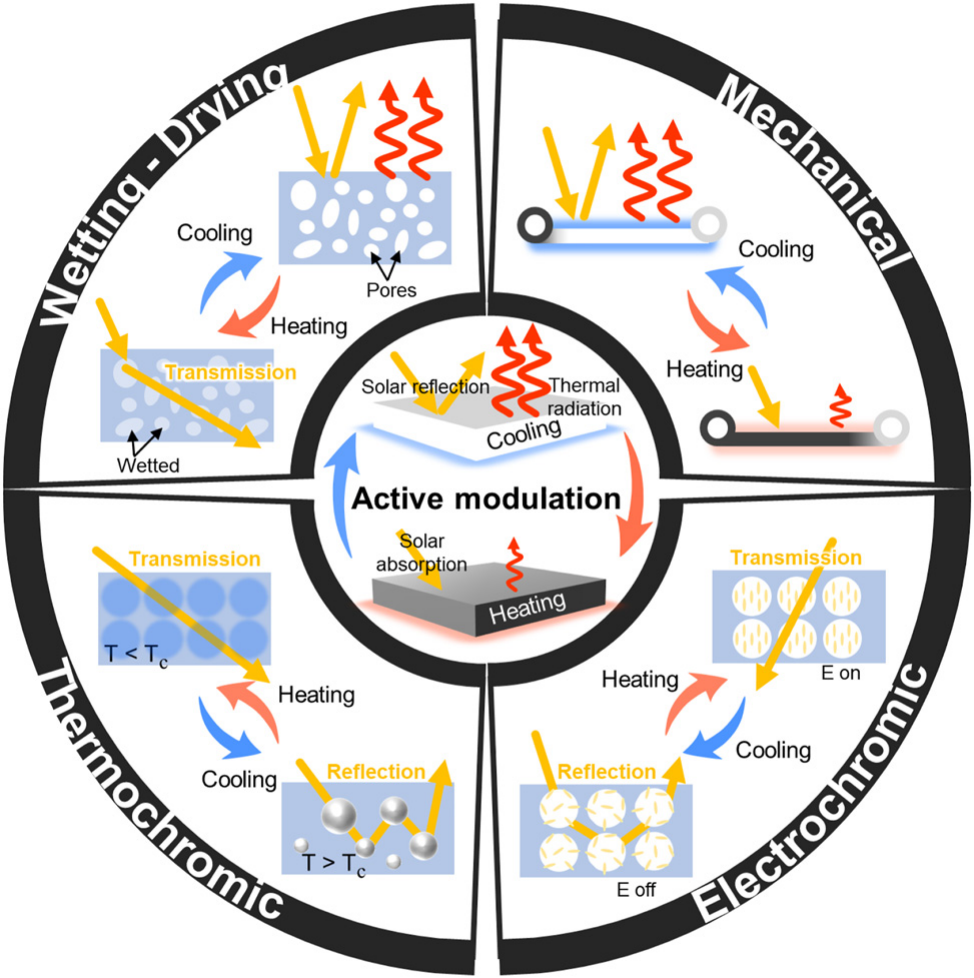
Summary of mechanisms of radiative thermal management, switching radiative cooling and solar heating.

While radiative cooling and solar heating offer energy-efficient thermoregulation strategies, their passive nature raises concerns regarding additional energy consumption [4]. This is because they can unintentionally result in undesired cooling during cold winters or heating during hot summers, which compels us to use additional energy. Moreover, in the face of recent dynamically changing abnormal climate conditions [5–7], passive radiative cooling and solar heating cannot respond quickly. It necessitates the adoption of much more adaptable radiative thermal management technologies [8–10]. In response to these demands, novel approaches for switchable radiative thermal management have been developed using mechanisms based on wetting [11–15], mechanical [16–21], thermochromic [22–25], electrochromic [26–29], and more (Figure 1). These technologies have demonstrated their switchability between sub-ambient radiative cooling and above-ambient solar heating by adjusting optical properties in the solar wavelength or infrared (IR) wavelength region, resulting in efficient thermal management with minimal energy consumption. However, for practical usage, these switchable thermal radiative management technologies have remaining challenges [10, 30], [31], [32].

In this review, we introduce the basic concept of realizing switchable radiative cooling and heating by optical property regulation. We also summarize recent advancements in switchable radiative cooling and heating technologies depending on the switching mechanisms. Furthermore, we also delve into the challenges of present switchable radiative thermoregulation technologies and offer directions for future research in the development of switchable radiative cooling, considering the need for practical applications.

## 2 Basic concept of switchable radiative cooling and solar heating

In the context of radiative thermal management, the solar reflectivity (*R*_*solar*_) within the solar wavelength range (0.3–2.5 μm) and the long-wave infrared (LWIR) emissivity (*ε*_*LWIR*_) within the atmospheric window range (8–13 μm) are the most important parameters, regulating the solar irradiance and the thermal emission into space, respectively [33].

*R*_*solar*_ is a measure of how much solar radiation is reflected by a material with respect to the incident solar intensity, formulated as follows:

(1) $$R_{\mathrm{solar}}=\frac{\overset{2.5\;\;\mu m}{\underset{0.3\;\;\mu m}{\int }}I_{\mathrm{solar}}\left(\lambda \right)R\left(\lambda \right)d\lambda }{\overset{2.5\;\;\mu m}{\underset{0.3\;\;\mu m}{\int }}I_{\mathrm{solar}}\left(\lambda \right)d\lambda }$$

where *λ* is the wavelength, $I_{\mathrm{solar}}\left(\lambda \right)$ is generally AM 1.5G global solar intensity spectrum, and *R*(*λ*) is the spectral reflectance of the surface of the materials.

On the other hand, *ε*_*LWIR*_ refers to a property that describes how well a material emits electromagnetic waves in the LWIR wavelength range compared to a blackbody at room temperature, defined as follows:

(2) $$\varepsilon_{\mathrm{LWIR}}=\frac{\overset{13\;\mu m}{\underset{8\;\mu m}{\int }}I_{BB}\left(T,\lambda \right)\varepsilon \left(\lambda \right)d\lambda }{\overset{13\;\mu m}{\underset{8\;\mu m}{\int }}I_{BB}\left(T,\lambda \right)d\lambda }$$

where *I*_*BB*_(*T*, *λ*) is the spectral intensity of the blackbody at the temperature of the emitter (*T*) and *ε*(*λ*) is the spectral emissivity of the emitter.

**Figure 2:**
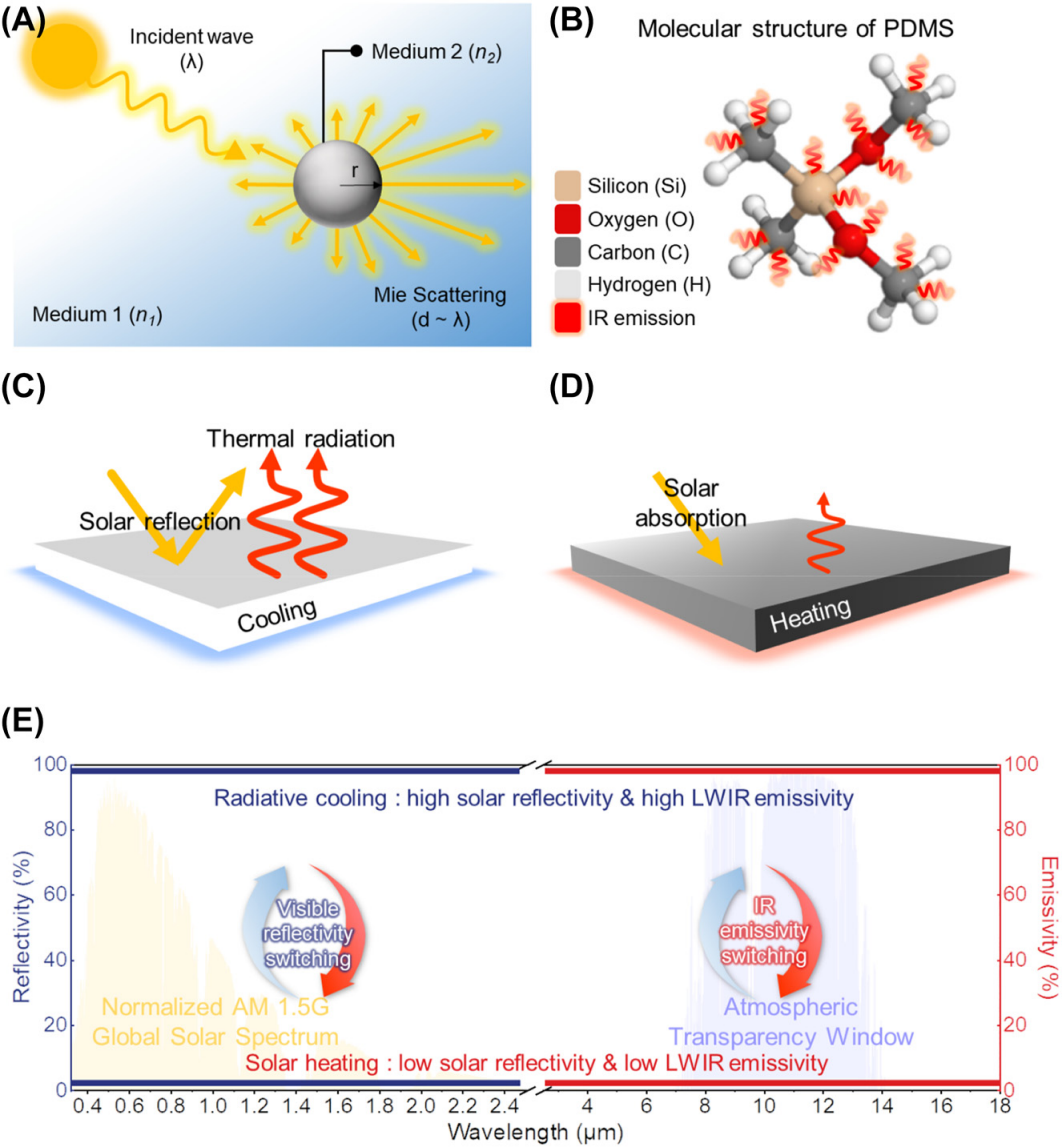
Basic concepts regarding to switchable radiative cooling and solar heating. (A) Graphical illustration of Mie scattering describing electromagnetic wave incident to medium. (B) Illustration of the molecular structure of PDMS having LWIR emissive molecular bonds and showing high *ε*_*LWIR*_. (C) Schematic of radiative cooling mode. (D) Schematic of solar heating mode. (E) The ideal spectral properties of radiative cooling and solar heating with required switchability in the solar spectrum and atmospheric transparency window to achieve radiative thermal management.

As is evident from the definition, designing *R*_*solar*_ and *ε*_*LWIR*_ plays a crucial role in achieving the desired radiative cooling or heating performance of the material. This necessitates a basic understanding of the mechanisms behind the two important optical properties. Firstly, the primary governing principle in determining *R*_*solar*_ is the scattering of incident sunlight induced by Mie scattering [11, 15, 23]. As depicted in Figure 2A, Mie scattering particularly occurs when the scale (*r*) of the scattering medium (such as spheres, infinite cylinders, and other geometries) is similar to the wavelength of an incident wave (*λ*) and when the refractive index of the scattering medium (*n*_*2*_) is different from that of the surrounding medium (*n*_*1*_) [34]. In general, structures with scatterers of similar size to the wavelength of incident sunlight exhibit high Mie scattering efficiency (*Q*_*scat*_) [35, 36], leading to increased reflectivity of sunlight. Since solar intensity is especially strong in the visible spectrum (380–700 nm) and the near-infrared (NIR) spectrum (1000–2500 nm), the introduction of nano and microstructures is important [15, 30, 37], [38], [39], [40].

Secondly, the factor determining *ε*_*LWIR*_ is the intrinsic molecular structure of the material [11, 39, 41]. It is validated that molecular bonds such as C–O–C (1260–1110 cm^−1^), C–OH (1239–1030 cm^−1^), and C–F_3_ (1148 cm^−1^) induce strong vibrational energy resonance within the LWIR wavelength range and thus result in the IR-absorbing property of the material and high *ε*_*LWIR*_. For instance, polydimethylsiloxane (PDMS), a popular material with high IR emissivity [42–44], has molecular bonds such as Si–O (1019 cm^−1^) and Si–CH_3_ (873 cm^−1^), which contribute to its high *ε*_*LWIR*_ (Figure 2B). Also, the presence of C–F (1234–1279 cm^−1^), C–H (855–976 cm^−1^), and C–H_2_ (812–840 cm^−1^) in polyvinylidene fluoride (PVDF), polytetrafluoroethylene (PTFE) and polymers contributes to its high *ε*_*LWIR*_, making them promising materials for radiative cooling [23, 37, 45], [46], [47], [48].

Another factor that determines *ε*_*LWIR*_ is the material’s crystal structure. For instance, vanadium oxide (VO_2_) exhibits two distinct phases based on its crystal structure: the metallic and insulating phases [49, 50]. Metallic-phase VO_2_ tends to be IR-emissive, while insulating-phase VO_2_ is known for its high IR transmittance, resulting in low *ε*_*LWIR*_. Similarly, metals like gold generally have high reflectivity for electromagnetic waves due to their crystal structure, which leads to the low *ε*_*LWIR*_ [51]. In radiative cooling mode, it is essential to block solar irradiance while allowing efficient thermal emission [52]. Therefore, high *R*_*solar*_ and high *ε*_*LWIR*_ are required (Figure 2C). Conversely, sunlight absorption/transmission is necessary for solar heating mode while minimizing thermal emission [16, 18, 19], necessitating low *R*_*solar*_ and low *ε*_*LWIR*_ (Figure 2D).

By modulating these two optical properties through diverse switching mechanisms, radiative thermal management devices can achieve reversible switching between cooling mode with high *R*_*solar*_ and high *ε*_*LWIR*_ and heating mode with low *R*_*solar*_ and low *ε*_*LWIR*_ (Figure 2E). The details of this switching principle will be discussed in the following section, categorized by the switching mechanisms.

## 3 Various mechanisms for switchable radiative cooling

### 3.1 Wetting/drying switching mechanism

**Figure 3:**
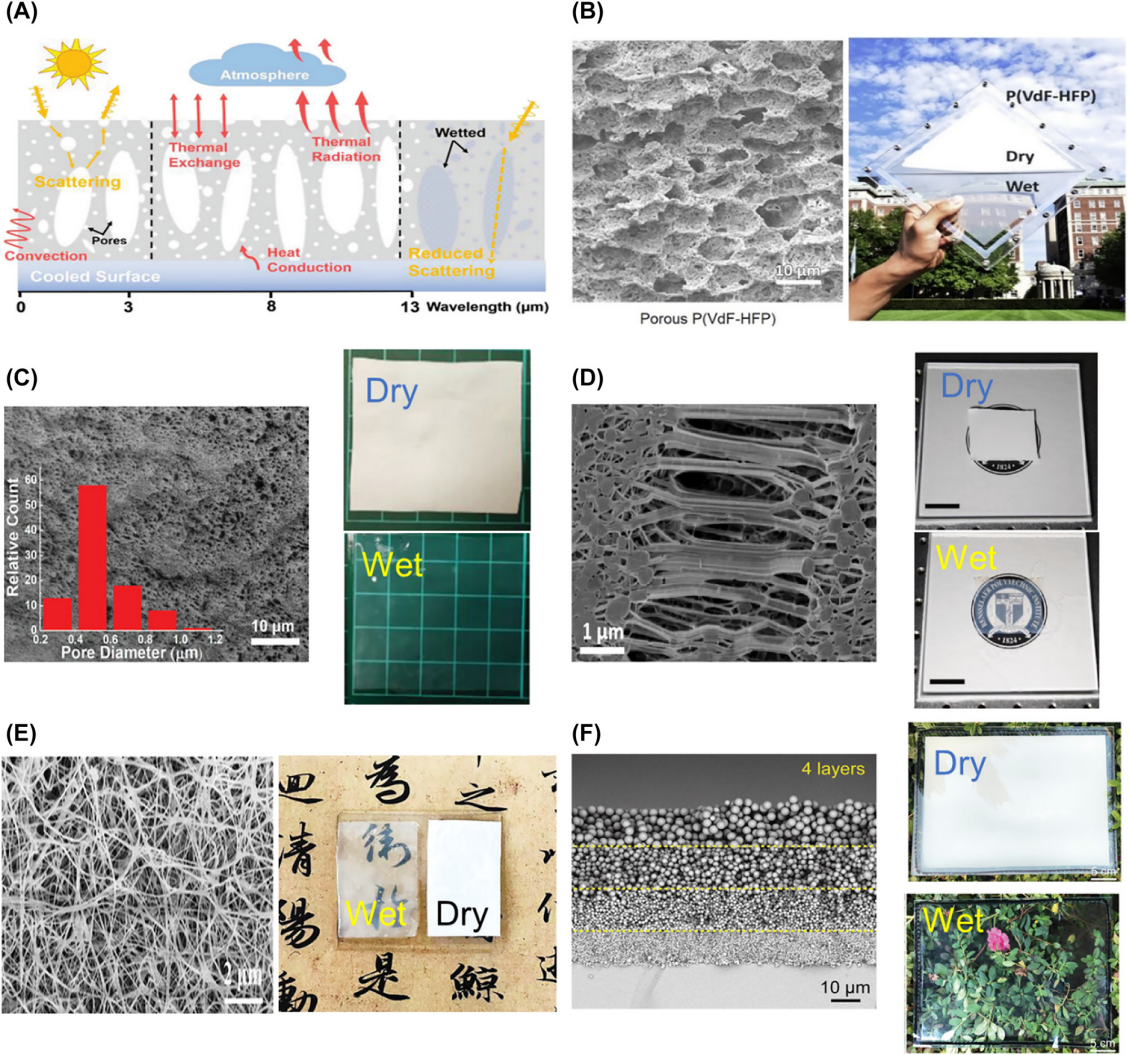
Radiative thermal management using a wetting/drying mechanism. (A) System concept of regulating sunlight scattering with nano/microporous structure and refractive index-matched liquid for thermal management: radiative cooling mode at left and middle and solar heating mode at right side. Reproduced with permission from Ref. [11]. Copyright 2022 John Wiley and Sons. (B) Scanning electron microscope (SEM) micrograph of porous P(Vdf-HFP) (left, scale bar 10 μm) and photograph of the PPC under dry and wet state (right). Reproduced with permission from Ref. [12]. Copyright 2019 Elsevier. (C) SEM image of porous HPC structure (left, scale bar 10 μm) and optical images of HPC under dry and wet state (right). Reproduced with permission from Ref. [11]. Copyright 2022 John Wiley and Sons. (D) SEM image of porous PTFE layer (left, scale bar 1 μm) and real images under dry and wet state (right). Reproduced with permission from Ref. [14]. Copyright 2022 Elsevier. (E) SEM image of porous electrospun bacterial cellulose nanofibers film (left, scale bar 2 μm) and photo of film in wet and dry state (right). Reproduced with permission from Ref. [13]. Copyright 2023 Wiley Johns and Sons. (F) SEM image of 4-layers hierarchical SiO_2_ particle porous coating (left, scale bar 10 μm) and optical images of dry and wet state (right). Reproduced with permission from Ref. [15]. Copyright 2022 Wiley Johns and Sons.

One of the simplest cooling/heating modes switching methods is to immerse nanostructured porous materials with a refractive index similar to the refractive index of the material in a liquid. Porous materials with nano/micropores in the range of 0.1–10 μm exhibit efficient scattering of light in the solar wavelength range (0.4–2.5 μm) due to the high refractive index contrast (*Δn* = *n*_*material*_ – *n*_*air*_) at the material-pore boundary, resulting in high *R*_*solar*_ and a bright white appearance. It enables a cooling mode with high spectral reflectivity in the solar wavelength range (0.4–2.5 μm). This can be explained by the fact that through Mie scattering, pores with sizes around 0.1 μm scatter shorter wavelengths, while pores with sizes up to ∼10 μm scatter longer wavelengths effectively [53]. However, when these porous materials are filled with a liquid having a refractive index similar to that of the material, replacing the air in the pores with the liquid, the refractive index contrast (*Δn* = *n*_*material*_ – *n*_*liquid*_) is greatly reduced, almost eliminating the refractive index contrast (Figure 3A) [11]. The drastic decrease in refractive index contrast leads to a decrease in Mie scattering efficiency, resulting in low *R*_*solar*_ (high solar transmittance) and a transition to a heating mode where solar irradiation is well transmitted/absorbed. Therefore, the radiative cooling materials with nano/microporous structures can exhibit optically switchable behavior, demonstrating high *R*_*soalr*_ and high *ε*_*LWIR*_ in the dry state (cooling mode) for radiative cooling and high solar transmittance in the wet state (heating mode) for solar heating which can efficiently regulate radiative thermal management.

Mandal et al. [12] demonstrated radiative thermal management by refractive index matching through wetting P(Vdf-HFP) porous polymer coating (PPC) with the refractive index-matched liquid for the first time. As depicted in Figure 3B, the PPC exhibited a high *R*_*solar*_ in the cooling mode, while in the heating mode, it exhibited a high solar transmittance (*T*_*solar*_) of 0.94. Additionally, in the dry state, the porous structure of PPC with nano/micropores in the range of 0.1–10 μm leads to efficient Mie scattering in the solar wavelength range, resulting in low solar transmittance (*T*_*solar, dry*_ = 0.2) due to a significant refractive index contrast at the P(Vdf-HFP)-pore boundary (*Δn = n*_*P(Vdf-HFP)*_ *– n*_*air*_ ∼ 1.40–1 = 0.4). However, when P(Vdf-HFP) PPC is filled with isopropanol (*n* ∼ 1.38), a sudden decrease in refractive index contrast (*Δn* = 1.40–1.38 = 0.02) leads to refractive index matching, resulting in an increased solar transmittance (*T*_*solar*, wet_ = 0.94) due to drastic reduction of the light scattering. The significant advantage of their PPC approach is that the modulation of solar transmittance can be applied to various other polymers using the same approach. They also created a switchable radiative cooling roof using PTFE in the same PPC form, which exhibited *R*_*solar*_ = 0.95 in the dry state and achieved *ΔT*_*solar*_ *= T*_*solar, dry*_ *– T*_*solar, wet*_ ∼ 0.42 upon filling. Temperature measurement experiments were conducted, demonstrating sub-ambient radiative cooling of approximately 3.3 °C in the cooling mode and above-ambient solar heating of approximately 21.4 °C in the heating mode under solar intensity of 1043 W m^−2^.

Similarly, Fei et al. [11] achieved practical switchable thermal and optical regulation by creating a unique hierarchical polymer porous coating (HPC) structure with vertically aligned microscale pores within a nanoscale porous matrix using PVDF/cellulose acetate (CA) polymeric network. Thanks to the hierarchical arrangement of nanoscale and microscale pores, it exhibits efficient scattering due to the high refractive index contrast between the polymer and air, as well as the effective scattering resulting from the comparable pore sizes to the solar wavelength range. This leads to high *R*_*solar*_ (∼96.6 %) and excellent radiative cooling performance, aided by the intrinsic high *ε*_*LWIR*_ (>96 %) of the polymer. Wetting HPC structure with refractive index-matched liquids exhibits high *T*_*solar*_ (∼86.6 %), transitioning to solar heating mode. Because the speed of state transition and operating temperature is also crucial for efficient thermal regulation, the authors enhanced the hydrophilicity of the polymeric chain in the HPC through Poly(ethylene glycol)-400 treatment, which enriches hydroxyl groups, and used IPA as the refractive index-matched liquid due to its low boiling point. They demonstrated that optical switching could be accomplished within 1 min by employing a rubber blower in the system.

Also, Wang et al. [14] created a PTFE-SS multilayer structure (Figure 3D) by combining a porous PTFE layer with a cermet-based spectrally selective absorber made of nickel-embedded alumina. They harnessed the phenomenon in which the porous PTFE layer exhibits high *R*_*sola*r_ in the dry state and becomes transparent when wet, enabling radiative thermal management. They encapsulated a 5-layer PTFE between two borosilicate substrates, achieving a performance of *ΔT*_*solar*_ = 0.59. By utilizing the selective absorber, they maximized solar absorption in the heating mode. Through temperature measurement experiments using a solar simulator, they achieved a cooling mode performance of 30.5 °C and a heating mode performance of 76.9 °C under 1-solar irradiation. Additionally, Shi et al. [13] created a similar polymer-based radiative cooling technology based on biosynthetic bacterial cellulose (Bio-RC, Figure 3E). They utilized porous nanofibers (BC nanofibers) made through an electrospinning process and embedded silica microparticles. It exhibited a *R*_*solar*_ of 95.3 % in the dry state, while in the wet state, it exhibited a *T*_*solar*_ of 70 %. By enhancing bacterial intrinsic *ε*_*LWIR*_ of cellulose through the phonon-polariton resonances of Si–O bond, they achieved an *ε*_*LWIR*_ of 93.4 %. Figure 3E shows images of the Bio-RC film in the dry and wet states. Leveraging the switchable optical modulation properties of the Bio-RC film, they combined it with semi-transparent solar cells to create the roof of a model house, both enabling enhancement of power conversion efficiency of solar cells and smart radiative thermal management.

In addition, Zhang et al. [15] developed a thermal management device with self-adaptability (STMD) that enables switchable solar heating and radiative cooling by utilizing a porous layer of SiO_2_ nano/microparticles and a refractive index-matched liquid. SiO_2_ particles are known to have low solar absorption in the solar wavelength range and exhibit high *ε*_*LWIR*_. As a result, the SiO_2_ porous layer in its dry state demonstrates excellent radiative cooling performance (*T*_*solar, dry*_ = 0.2). Furthermore, when immersed in carbon tetrachloride, a refractive index-matched liquid, the refractive index matching renders it highly transparent in the solar wavelength range, inducing a solar heating mode. Additionally, the authors designed the structure of the porous coating based on the principle that efficient scattering occurs when the incident light is comparable to the particle size, as explained by Mie scattering theory. Through finite-difference time-domain (FDTD) simulations, they calculated the scattering efficiency within the solar wavelength range based on the size of the SiO_2_ particles and observed that the spectral peak of the scattering efficiency red-shifted with increasing particle size. Based on this, they created a multi-layered SiO_2_ particle porous coating structure with hierarchical particle sizes ranging from 500 nm to 3 μm (Figure 3F), maximizing the *R*_*solar*_ while maintaining high *T*_*solar*_ in the wetted state, optimizing the solar modulation capability. By encapsulating SiO_2_ particle coating within a closed chamber, STMD can be dried or wetted by a refractive index-matched liquid with a fast switching speed (∼2 min). In hot weather, the liquid evaporates, causing it to separate from the porous coating and facilitate radiative cooling. In cold weather, the evaporated liquid condenses back into a liquid state, immersing the porous coating and enabling advanced self-adaptive radiative thermal management through cooling and heating switching. In outdoor temperature measurement experiments under an average solar irradiance of 850 W m^−2^, the STMD achieved sub-ambient cooling of 5 °C in the cooling mode and above-ambient heating of 10 °C in the heating mode, saving 55 and 51 MJ m^−2^ of energy, respectively.

### 3.2 Mechanical switching mechanism

Mechanical stimuli such as flipping, rotating, compressing, and stretching are among the most widely used methods to manipulate the state of materials [16–21]. The mechanical approach shows great promise as a method for switching between radiative cooling mode and solar heating mode, offering the most variable structural system designs with low energy consumption. Mechanical mechanisms for operating radiative thermal management systems include flipping Janus films with one side exhibiting radiative cooling properties and the other with solar heating characteristics, rolling structures where radiative cooling and solar heating layers are separated on side-by-side, actuating layers sandwiched between cooling and heating layers to enable rolling for switching upon actuation, control of material porosity through stress or strain to modify scattering characteristics and *R*_*solar*_, and the use of nanophotonic structures that gratings are variable under strain, offering flexibility and versatility in achieving radiative thermal management.

**Figure 4:**
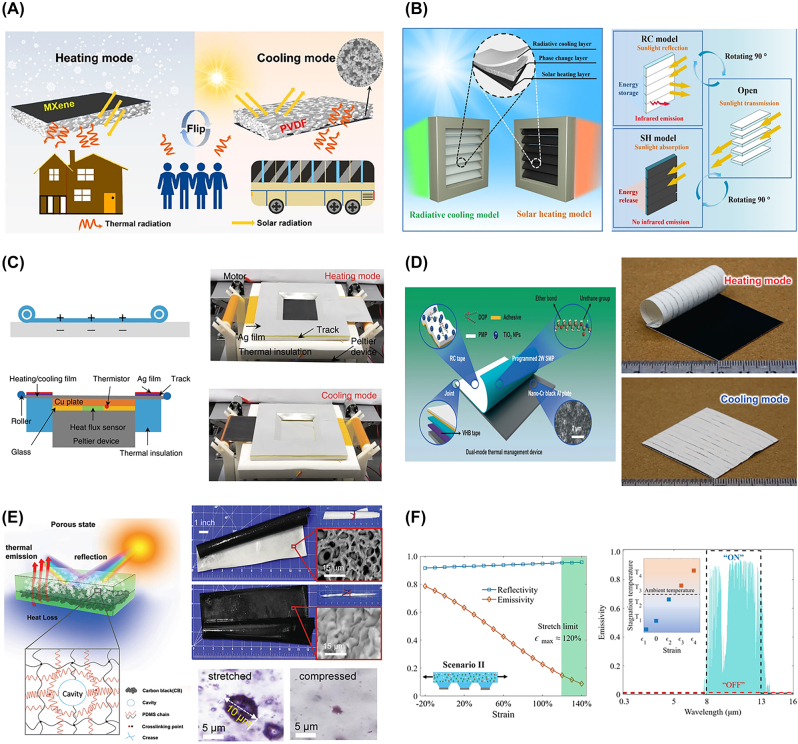
Radiative thermal management using mechanical mechanism. (A) Schematic diagram of the dual-mode film with one side for MXene-based solar heating and the other for PVDF film-based radiative cooling. Reproduced with permission from Ref. [18]. Copyright 2023 American Chemical Society. (B) Structure of louver-like radiative thermal management device with a rotating blade having radiative cooling and solar heating layer on each side. Reproduced with permission from Ref. [19]. Copyright 2023 American Chemical Society. (C) Illustration of the radiative thermal management system (left) altering cooling and heating modes by rolling the film and its real images (right). Reproduced with permission from Ref. [16] under the terms and conditions of the Creative Commons Attribution CC BY 4.0 license. Copyright 2020 Li et al. published by Springer Nature. (D) The structure of the radiative thermal management device, in which the actuating layer is sandwiched between the cooling and heating layer (left) with optical images under heating and cooling modes (right). Reproduced with permission from Ref. [20] under the terms and conditions of the Creative Commons Attribution CC BY 4.0 license. Copyright 2022 Zhang et al. published by Springer Nature. (E) Schematic of the mechanically switchable layer structure composed of the metastable cavity (left) and its real SEM image (top right, scale bar 15 μm) in opaque and transparent modes. Formation of cavity depending on the mechanical stress (bottom right, scale bar 5 μm). Reproduced with permission from Ref. [21] under the terms and conditions of the Creative Commons CC BY license. Copyright 2020 Cui et al. published by John Wiley and Sons. (F) Reflectivity and emissivity of the structure depending on the applied strain (left) and schematic of toggling radiative cooling by modulation of *ε*_*LWIR*_ (right). Reproduced with permission from Ref. [17] under the terms and conditions of the Creative Commons Attribution CC BY 4.0 license. Copyright 2020 Liu et al. published by Springer Nature.

Shi et al. [18] created a dual-mode Janus layer by placing a solar heating layer, an MXene film, onto a radiative cooling layer composed of a porous PVDF layer (Figure 4A). The cooling side exhibits high values of *R*_*solar*_ (96.7 %) and *ε*_*LWIR*_ (96.1 %), while the heating side shows high solar absorption (75.7 %) and low *ε*_*LWIR*_ (11.6 %). Additionally, due to the excellent electrical conductivity of MXene, the dual-mode structure allows for additional heating through Joule heating. The dual-mode film enables easy switching between cooling and heating modes by simply flipping it, eliminating the need for complex systems. In outdoor temperature measurement experiments with solar irradiance below 500 W m^−2^, the radiative cooling performance achieved sub-ambient cooling of 10.5 °C during the daytime and 11.1 °C during the nighttime. Through flipping, the dual-mode Janus film enables solar heating and allows for the utilization of Joule heating. This additional feature permits the fine-tuning of heating power within the range of 1–5 V input voltage, enhancing the precision of thermal management. Another Janus structure is presented by Yang et al. [54], wherein a Janus-structured aerogel composed of MXene-cellulose nanofiber (CNF)/CNF reveals different photothermal properties depending on the exposed side. When the CNF layer is exposed, it exhibits a high *R*_*solar*_ due to its low solar absorption and porous structure with high *ε*_*LWIR*_, leading to efficient radiative cooling. Conversely, when the MXene-CNF layer is exposed, it achieves solar heating thanks to its high solar absorption, making it suitable for temperature-adaptive smart roofs. Using the Janus structure-based house modeling, they maintained above 25 °C in winter and under 30 °C in hot summer.

Similarly, Tao et al. [19] designed a component with a sandwich structure consisting of a radiative cooling emitter and a solar heating film with an interlayer of phase change material arranged as blades of louver. In this work, the blade rotation allows for the switching between radiative cooling mode and solar heating mode (Figure 4B), while the phase change material enables steady thermal management by storing and releasing latent heat. For radiative cooling, they utilized a fluorocarbon resin comprising PVDF and acrylic resins, which exhibits high *ε*_*LWIR*_, along with a silver–aluminum alloy coating as a solar reflector with high reflectivity, offering enhanced corrosion and seasonal resistance. As for solar heating, a structure comprising a solar absorption layer and an anti-reflection layer was employed. In radiative cooling mode, the emitter effectively radiates heat while the phase change material stores heat in latent heat, thereby cooling the space. In solar heating mode, the heating film fully absorbs solar radiation, while the phase change material efficiently releases latent heat to heat the space effectively. To assess the performance of the device, they conducted outdoor temperature measurement experiments with the louver-like radiative thermal management device from 9:00 to 18:00. The results demonstrated that switchability and the presence of the phase change material of device provide significantly more stable temperature regulation against the daily ambient temperature fluctuation, achieving temperature difference between cooling and heating mode of ∼25 °C.

Li et al. [16] also developed a radiative thermal management device using a thin film polymer. Laterally separated two composite films for cooling and heating are switched by rolling, using two rotary machines positioned at each end. They deposited a 300 nm thick silver film on a PI film as radiative cooling material to reflect solar radiation. Then, they layered it with PDMS possessing high *ε*_*LWIR*_, resulting in a high *R*_*solar*_ of 97.3 % and *ε*_*LWIR*_ of 94.1 %. For the heating layer, they uniformly deposited copper and copper oxide nanoparticles on a Zn film for the heating material, achieving solar heating properties of 93.4 % solar absorption and 14.2 % *ε*_*LWIR*_. As shown in Figure 4C, they created a dual-mode system and conducted outdoor experiments. During the experiments, the heating and cooling modes switched through motor rotation in 15 min per switching cycle. In the heating mode, they measured a positive heat flux of over 400 W m^−2^, while in the cooling mode, they recorded a negative heat flux of up to −70 W m^−2^, confirming the thermal management performance of the device.

Zhang et al. [20] proposed a thermal management device with automatically switching cooling and heating modes featuring a zero-energy characteristic achieved through a temperature-sensitive actuating layer. They utilized an aluminum plate coated with Chromium (Cr) nanopowder for the heating layer, known for its high sunlight-heat transformation efficiency due to plasmon resonance. As for the radiative cooling material, they created a stretchable radiative cooling tape (RC tape) in a composite form, combining dioctyl phthalate (DOP)-modified poly(4-methyl-1-pentene) (PMP) with rutile titanium dioxide nanoparticles (TiO_2_ NPs). The significant difference in refractive indices between PMP and TiO_2_ NPs resulted in effective sunlight scattering efficiency (*Δn* = *n*_*TiO2*_ – *n*_*PMP*_ > 0.93) and high *R*_*solar*_ > 90 %. Using a wide range of particle sizes (0.1–0.9 µm) based on FDTD Mie scattering simulation ensured efficient scattering across the entire solar spectrum. Moreover, the DOP-modified PMP and TiO_2_ NPs exhibited prominent peaks in the infrared spectral region, leading to high *ε*_*LWIR*_ (∼96 %) and excellent radiative cooling performance. To achieve auto-switchable properties, they sandwiched a temperature-sensitive actuating layer with reversible shape memory (composed of polytetrahydrofuran (PTHF), polycaprolactone (PCL), and hexamethylene diisocyanate (HDI)) between the cooling and heating layers. As depicted in Figure 4D, the actuating layer undergoes reversible melting-crystallization triggered by temperature changes, resulting in longitudinal contraction when heated and expansion when cooled, thereby bending the RC tape to automatically switch between cooling mode (expanded RC tape, heating layer concealed) and heating mode (shrunk RC tape, heating layer visible). The researchers evaluated the thermal management performance of the device by measuring the heat flux in both heating and cooling modes under outdoor conditions with solar irradiation of 850 W m^−2^ or higher. The device demonstrated 92 % solar-thermal conversion efficiency and a 126 ± 41.6 W m^−2^ cooling power performance.

In contrast to mechanically flipping or rotating the two separated cooling/heating layers, as mentioned earlier, Zhao et al. [21] developed a thermal management device using an optically switchable layer with a mechanically reversible cavity in PDMS, enabling it to transition between a transparent solid state with low *R*_*solar*_ and a highly porous state with high *R*_*solar*_ through mechanical stimuli such as stretching and compressing. By emulsion polymerization of PDMS precursor with water, the curing process induced evaporation of water droplets, creating negative pressure and leading to metastatic creases. Figure 4E shows that these creases exist in an extended state when the layer is stretched, resulting in a highly porous and opaque cooling mode. Conversely, the creases disappear when the layer is compressed, and the porosity diminishes, creating a solid and transparent solar heating mode. The switchable layer was placed above carbon black particles (CBPs) that functioned as the solar absorption layer, forming a bifunctional bilayer. The heating layer beneath could be concealed or revealed depending on mechanical stretching or compression, enabling switchable cooling and heating modes. In cooling mode, the bilayer exhibited high *R*_*solar*_ (∼93 %) and around 94 % of *ε*_*LWIR*_. It could absorb approximately 95 % of sunlight through the absorption layer in heating mode. Through outdoor temperature measurement experiments, they demonstrated that during cold seasons, the device could achieve heating of approximately 8 °C at an ambient temperature of around ∼10 °C) under solar intensity of approximately 795 W m^−2^. In hot seasons with an ambient temperature of approximately 35 °C and solar irradiance of 768 W m^−2^, the device achieved sub-ambient cooling of around 5 °C. Notably, besides the excellent thermal management performance of the bilayer, the researchers highlighted the simplicity of the fabrication, which allows it to be applied to various substrates using painting or casting techniques.

Mechanical mechanisms are not limited to modulation within the solar spectrum but extend to LWIR wavelength ranges. Liu et al. [17] theoretically proposed the dynamic tuning of emissivity based on mechanical stress/strain using deformable nanophotonic structures made of PDMS in response to dynamic temperature variations. As depicted in Figure 4F, they created a one-dimensional square grating structure on PDMS mixed with silicon carbide (SiC), silicon nitride (Si_3_N_4_), and boron nitride (BN) nanoparticles and then deposited a silver film on top of it. Mechanical deformation changes the thickness of the nanoparticles-PDMS layer and the period of grating, which induces adjustment of *ε*_*LWIR*_ in the range of 23 % (strain ∼ 120 %) to 79 % (no strain), enabling the regulation of radiative cooling for thermal management through mechanical stress/strain. They conducted thermal performance calculations with an ambient temperature set at 25 °C. When the strain was below 20 %, the net cooling power was positive, leading to sub-ambient cooling of up to 6.1 °C. However, for strain levels exceeding 20 %, negative radiative cooling power resulted in heating, with temperatures rising by up to 14.76 °C above ambient. Furthermore, Li et al. [55] implemented radiative thermal regulation by leveraging the continuous changes in the optical properties of a thermoplastic polyurethane (TPU) nanofiber membrane when mechanical deformation is applied to alter its pore density and thickness. In its undeformed state, TPU exhibits high *R*_*solar*_ due to its inherent high *ε*_*LWIR*_ and porosity, enabling effective radiative cooling. However, when subjected to significant strain, an increase in pore size reduces scattering efficiency, and a decrease in membrane thickness leads to lower *R*_*solar*_ and *ε*_*LWIR*_, allowing for solar heating. Through outdoor temperature evaluation experiments, they confirmed sub-ambient cooling of 10 °C at 0 % strain and above-ambient heating of 9.5 °C at 80 % strain.

### 3.3 Thermochromic switching mechanism

**Figure 5:**
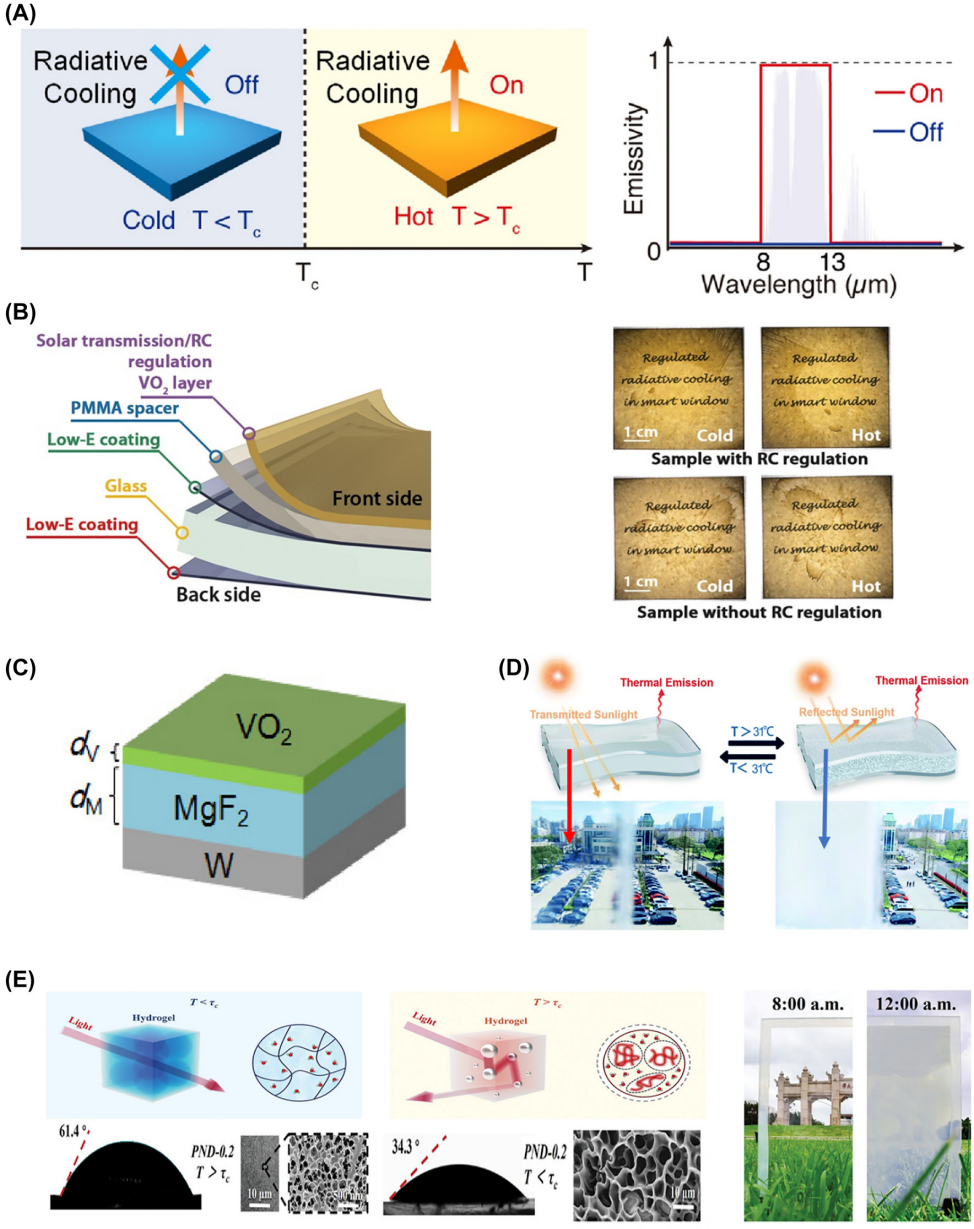
Radiative thermal management using thermochromic mechanism. (A) Schematic of the concept of thermal management modulating *ε*_*LWIR*_ of thermochromic materials depending on temperature. Reproduced with permission from Ref. [24] under the terms and conditions of the Creative Commons Attribution CC BY 4.0 license. Copyright 2018 Ono et al. published by Optica Publishing Group. (B) Illustration of VO_2_-based RCRT window regulating thermal emission (left) and images of the RCRT window with thermal control (above) and the VO_2_ window without RC control (below) at both low and high temperatures. Reproduced with permission from Ref. [61]. Copyright 2021 American Association for the Advancement of Science. (C) Graphical illustration of LWIR modulating photonic structure composed of VO_2_/MgF_2_/W. Reproduced with permission from Ref. [24] under the terms and conditions of the Creative Commons Attribution CC BY 4.0 license. Copyright 2018 Ono et al. published by Optica Publishing Group. (D) Conceptual image of thermochromic PNIPAm hydrogel regulating reflection of sunlight (top) and optical images under different temperatures, inducing transparent and opaque state (bottom). Reproduced with permission from Ref. [23]. Copyright 2022 Royal Society of Chemistry. (E) Schematic of thermoregulating mechanism of PND hydrogel (left and middle) and real image under transparent and opaque state at different temperatures (right). Reproduced with permission from Ref. [22]. Copyright 2023 John Wiley and Sons.

Thermochromic materials, or temperature-responsive materials, are materials whose optical properties change with ambient temperature [56]. Thermochromic materials are promising for radiative thermal management, which undergo crystal/molecular structural phase transition in response to temperature variations, resulting in self-adaptive modulations in their *ε*_*LWIR*_ or *R*_*solar*_ [22, 23, 25, 57, 58]. For instance, vanadium oxide (VO_2_) exhibits insulator-metal phase transition at a critical temperature (*T*_*C*_, approximately ∼ 295 K) [49] at which it behaves as a low-loss insulator in the infrared wavelength range with low *ε*_*LWIR*_ at temperatures lower than *T*_*C*_. While at higher temperatures than *T*_*C*_, it acts as a plasmonic metal, absorbing infrared radiation and showing high LWIR absorptivity and emissivity (Figure 5A), turning radiative cooling on. By doping pure VO_2_ with materials like molybdenum (Mo) [59], tungsten (W) [24], or strontium (Sr) [60], researchers have been able to lower the transition temperature and combine it with solar reflective materials to manipulate thermal radiation through the atmospheric window, achieving effective thermal management [61]. Furthermore, recent studies have explored using certain hydrogels that display thermochromic properties due to changes in molecular chains and hydrogen bonding with water as a response to temperature variations [62–64]. Like the wetting mechanism, molecular structural of the hydrogels phase transition at the *T*_*C*_ changes refractive index contrast at the hydrogel–water interface, leading to modulation in *R*_*solar*_ due to alterations in scattering efficiency. The tunability of *ε*_*LWIR*_ and *R*_*solar*_ of such thermochromic materials with temperature demonstrates the potential for their utilization in self-adaptive radiative thermal management without additional energy consumption.

Wang et al. [63] employed W-doped VO_2_ nanoparticles with a room temperature *T*_*C*_ to create a Fabry–Perot resonator structure for a radiative cooling regulating thermochromic (RCRT) window (Figure 5B). This RCRT window exhibited a weak LWIR resonance at temperatures lower than *T*_*C*_, resulting in low *ε*_*LWIR*_. Conversely, at temperatures higher than *T*_*C*_, the VO_2_ phase transition enhanced LWIR resonance, leading to higher *ε*_*LWIR*_ and enabling up to 40 % LWIR modulation. The RCRT window remains transparent in the visible spectrum while allowing modulation in the NIR-LWIR range due to temperature changes, and this feature makes it suitable for radiative thermoregulating windows for buildings in various climates. Through energy consumption simulations for a building model with a total window area of 4636 m^2^ in various climatic conditions, they verified that the RCRT window achieved significant energy savings of up to 324.6 MJ m^−2^ annually. Additionally, Ono et al. [24] achieved self-adaptive LWIR modulation using a photonic structure composed of VO_2_/MgF_2_/W (Figure 5C). To enhance performance, they placed a spectrally selective filter made of Ge/MgF_2_ on the radiative cooler to block solar irradiation and provide high selective transmission in the LWIR wavelength range. Their thermal management evaluation involved simulating the temperature of the system, maintaining temperatures below approximately 298 K to operate below *T*_*C*_ and demonstrating cooling power of approximately 100 W m^−2^ at 313 K of exceeding *T*_*C*_.

Mei et al. [23] have implemented a self-adaptive smart thermal management using thermochromic poly(N-isopropyl acrylamide) (PNIPAm) hydrogel. The PNIPAm hydrogel experiences a phase change at a lower critical solution temperature (LCST) of approximately 31 °C. Below the LCST, the molecular chains of the PNIPAm hydrogel are relatively stretched, and strong hydrogen bonding with water molecules creates a hydration shell with a refractive index similar to that of water. This results in a transparent state in the solar spectrum. After the phase transition, the molecular chains curl up, releasing water molecules and creating boundaries between the polymer network and water. This leads to light scattering due to the refractive index contrast, resulting in a high *R*_*solar*_. The researchers created a sandwich-like structure to achieve radiative thermal modulation by placing the thermochromic PNIPAm hydrogel between transparent PVDF films with high *ε*_*LWIR*_. This PVDF-PNIPAm sandwich structure (PPSS) demonstrated a significant reflectivity modulation of 70 %, with a reflectivity of 12 % below the LCST and 82 % above it in the visible spectrum (300–780 nm). Furthermore, due to the presence of the PVDF film, the PPSS exhibited a high *ε*_*LWIR*_ (96 %). In outdoor temperature measurement experiments, PPSS below its LCST at ambient temperatures under 31 °C, under solar irradiance conditions of about 380 W m^−2^, maintained a solar-transparent state inducing solar heating with an increase in temperature of around 4.3 °C. At temperatures above its LCST, PPSS demonstrated sub-ambient cooling, lowering the temperature by approximately 1.8 °C at an ambient temperature of 40 °C under solar irradiance conditions of approximately 702 W m^−2^.

Similarly, Chen et al. [22] utilized a copolymerized hydrogel, P(NIPAm-co-DMAA) (PND) hydrogel, by incorporating the hydrophilic monomer N, N-dimethyl acrylamide (DMAA) into a PNIPAm hydrogel. They adjusted the content of the DMAA monomer to control the *T*_*C*_ of the thermochromic hydrogel, ranging from 32.5 °C to 43.5 °C, making it adaptable to various dynamic climates. The operational principle of the PND hydrogel is similar to PNIPAm; below the *T*_*C*_, the hydrophilic hydrogel’s refractive index closely matches that of water, resulting in high *T*_*solar*_. However, the PND molecular chains collapse after the phase transition, leading to hydrophobic domains that scatter light (Figure 5E). Furthermore, the pore diameter of the PND hydrogel above *T*_*C*_ is optimized for solar modulation, ranging from 60 to 535 nm, effectively scattering UV, visible, and NIR wavelength ranges. The researchers synthesized the PND hydrogel with a designed *T*_*C*_ of 40.26 °C and incorporated it between two quartz glass sheets separated by a silicone rubber spacer, creating a PND smart window for measuring thermal modulation performance. Through solar and LWIR spectral analysis at increasing temperatures of 35 °C, 40 °C, and 45 °C, they observed that the *T*_*solar*_ decreased from 84.54 % to 0.01 %, showing self-adaptive radiative thermal management.

In addition to the mentioned thermochromic materials, Liu et al. [65] utilized the thermal-induced phase separation between P(VDF-HFP) and an ionic liquid to create a thermochromic polymer coating (TPC). TPC functions by uniformly dispersing the ionic liquid within P(VDF-HFP) below its *T*_*C*_, allowing sunlight to pass through for solar heating. When the temperature exceeds *T*_*C*_, the ion–dipole interaction weakens, rendering the coating opaque. This transition, combined with its high *R*_*solar*_ and intrinsic molecular vibrations, enables effective radiative cooling. Moreover, the *T*_*C*_ of TPC can be adjusted simply by altering the composition of the ionic liquids, ranging from 33 to 43 °C.

### 3.4 Electrochromic switching mechanisms

Electrochromic materials possess the unique ability to adjust optical characteristics within specific wavelength ranges in response to electrical stimuli [66]. They have found extensive applications in various fields, including smart windows/glasses [67], electrochromic cells [68–70], and displays [71–73]. Due to their exceptional electro-optical modulation properties, including outstanding regulation of *R*_*solar*_ and *ε*_*LWIR*_, low-driving voltage, and ultrafast switching speeds [26], electrochromic materials can be effectively utilized in radiative thermal management. As a result, numerous research is underway to develop radiative thermal management technologies harnessing the potential of electrochromism [, 74, 75]. For instance, electrically controlled alignment of liquid crystals in polymer matrix or reversible electrodeposition of IR reflective metal on radiative cooler can be employed in thermal management devices.

**Figure 6:**
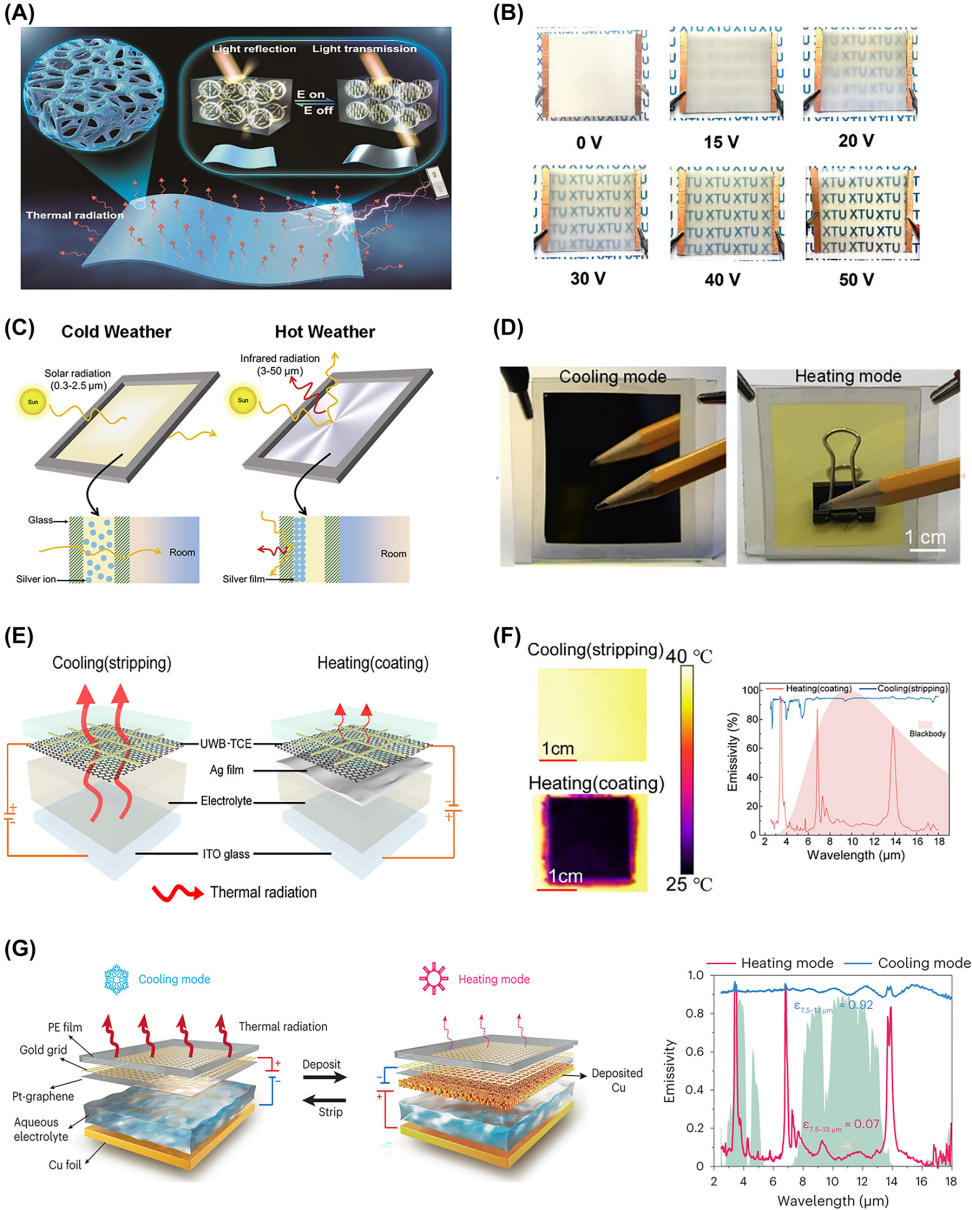
Radiative thermal management using electrochromic mechanism. (A) Schematic demonstration of light reflection regulation depending on the presence of an electric field in PDLC structure. Reproduced with permission from Ref. [26]. Copyright 2023 John Wiley and Sons. (B) Real images of transparency change induced by the magnitude of the voltage. Reproduced with permission from Ref. [26]. Copyright 2023 John Wiley and Sons. (C) Schematic diagram of thermal management device using electrodeposition of Ag. Reproduced with permission from Ref. [29] under the terms and conditions of the Creative Commons Attribution CC BY-NC-ND 4.0. Copyright 2022 Zhao et al. published by Elsevier. (D) Optical image of the device in Ag-deposited cooling mode and non-deposited heating mode. Reproduced with permission from Ref. [29] under the terms and conditions of the Creative Commons Attribution CC BY-NC-ND 4.0. Copyright 2022 Zhao et al. published by Elsevier. (E) Graphical illustration of ECD regulating thermal radiation with electrodeposition of Cu. Reproduced with permission from Ref. [27]. Copyright 2021 American Chemical Society. (F) IR images of ECD (left) and emissivity modulation spectrum (right) on cooling and heating modes. Reproduced with permission from Ref. [27]. Copyright 2021 American Chemical Society. (G) Schematic of ECD with enhanced emissivity modulation by Pt-modification. Reproduced with permission from Ref. [28]. Copyright 2023 Springer Nature.

Deng et al. [26] developed a switchable thermal management device utilizing electrochromic properties of liquid crystal dispersed in a porous polymer matrix in droplet form. The liquid crystal (E7) was mixed with UV-reactive monomers (CHMA/HFMA/PEGDA 600), which exhibit thermal emitting characteristics in the LWIR wavelength range. Upon UV irradiation, polymerization occurred, leading to phase separation between the liquid crystals and the polymer matrix, resulting in a polymer-dispersed liquid crystals (PDLC) structure. In the absence of an electric field, the PDLC film exhibited random liquid crystal alignment, causing scattering of sunlight and rendering the film opaque (*R*_*solar*_ ∼ 90 %). Moreover, certain chemical bonds (C–H, C–O, and C–F) in the polymer matrix efficiently radiated thermal energy in the LWIR wavelength range, facilitating effective radiative cooling (*ε*_*LWIR*_ = 92.6 %). When a voltage was applied to the film, liquid crystal microdroplets aligned uniformly along the electric field direction, eliminating scattering and allowing the film to become transparent (*T*_*solar*_ ∼ 55 % at 50 V), causing solar heating (Figure 6A). Experimental results using a well-designed temperature measurement system showed near-ambient cooling during daytime under solar irradiance of 400 W m^−2^ and sub-ambient cooling below that irradiance level. During sunless nighttime, sub-ambient cooling of 4.2 °C was observed. The most interesting aspect was the finely modulated luminous transmittance (*T*_*lum*_) within the range of 360–708 nm of the PDLC film according to voltage variations. At 0 V, *T*_*lum*_ was 0.19 %, increasing slightly to 2.5 % at 20 V, 16.2 % at 30 V, and reaching a maximum of 48.5 % at the saturation voltage of 50 V (Figure 6B). The elaborate *T*_*lum*_ modulation, leading to *R*_*solar*_ modulation, enables a practical radiative thermal management system that goes beyond simple switching between radiative cooling and solar heating modes, realizing fine thermoregulation.

Zhao et al. [29] have developed a dynamic glazing panel capable of switching between radiative cooling mode and solar heating mode through reversible silver electrodeposition. This dynamic glazing panel features a sandwich structure, with two transparent indium tin oxide (ITO)-coated glasses enclosing an electrolyte containing silver ions (Figure 6C). To enhance the *ε*_*LWIR*_ of the dynamic glazing panel in radiative cooling mode, an outer glass layer includes a transparent biaxially oriented polyethylene terephthalate (BoPET) film, raising the *ε*_*LWIR*_ of glasses to approximately 96 %. In Figure 6D, the dynamic glazing panel exhibits a low *R*_*solar*_ of approximately 17 % in solar heating mode. When a pulsed voltage of magnitude −2.5 V and frequency 7.0 kHz (current is about 11 mA) is applied, a silver film is deposited, transitioning it to radiative cooling mode with a high *R*_*solar*_ of about 89 %. With the sunlight absorber placed beneath the dynamic glazing panel, the thermal management performance of the device demonstrates sub-ambient cooling of up to 2 °C under solar irradiance below 600 W m^−2^ in cooling mode. In heating mode, it achieves approximately 24 °C of above-ambient heating performance. Similarly, Banerjee et al. [76] employed an electrochromic device using the conducting polymer poly[3,4-ethylenedioxythiophene]:tosylate (PEDOT:Tos) that exhibits IR electrochromism for radiative thermal management. By applying voltage to PEDOT:Tos, the oxidation/reduction of PEDOT occurs, leading to changes in charge carrier density, which alters IR reflectance and modulates the effective LWIR properties. They confirmed an IR emissivity of 77 % at −1.5 V and 54 % at 1.5 V.

Rao et al. [27] have engineered an electrochromic device (ECD) to maximize the radiative thermal management performance using a transparent working electrode composed of monolayer graphene, a gold microgrid, and polyethylene (PE), offering high electrical conductivity and transparency across a wide wavelength range of 0.2–20 μm (Figure 6E). The electrolyte of ECD consists of silver and copper ions along with IR-absorbing polar solvents. In the stripped state, it exhibits a high *ε*_*LWIR*_ of around 94 %, but upon applying a negative potential of approximately 1.5 V, a thin layer of low-emissivity silver and copper is electrodeposited, reducing *ε*_*LWIR*_ to about 20 %, resulting in excellent LWIR modulation performance. Furthermore, radiative cooling occurs in the stripped state when the ECD is used alongside an Ag back reflector due to the high *ε*_*LWIR*_ and *R*_*solar*_. In the deposited state, the random nanoparticles and nanoclusters on the working electrode serve as a solar absorber due to surface plasmon resonance within the solar spectrum, enabling solar heating and implementing radiative thermal management (Figure 6F). Sui et al. [28] enhanced the *ε*_*LWIR*_ modulation performance of a similar ECD structure by introducing Pt modification to the working electrode. In contrast to previous research, where Cu particles were large and sparsely deposited on bare graphene, Pt modification reduces the adsorption energy between Cu and the electrode surface, resulting in a denser and more continuous deposition that effectively reflects electromagnetic waves. This enhancement led to the highest reported *ε*_*LWIR*_ modulation, from 74 % to 85 %, among thermochromic/electrochromic thermal management devices (Figure 6G). They evaluated the radiative thermal management performance of the ECD by calculating annual energy savings in diverse climatic environments across 15 cities, revealing energy savings ranging from 5 to a maximum of 60 MBtu annually. They also demonstrated that dynamic radiative thermal management components offer more energy savings in climates with more severe daily and seasonal fluctuations.

## 4 Challenges and outlook

By employing various radiative cooling, solar heating materials and designing nano/micro-photonic structures and systems, it is possible to dynamically adjust the optical characteristics of materials, enabling smart and sustainable radiative thermal management. The structural design of radiative thermal management devices primarily focuses on modulating spectral reflectivity within the solar spectrum wavelengths or emissivity in the range of LWIR wavelengths using various switching mechanisms. The first approach involved adjusting the scattering efficiency and *R*_*solar*_ through wetting and drying the nano/microstructure of radiative cooling materials with refractive index-matched liquids. It enables efficient radiative thermal management, thanks to intrinsic radiative cooling performance of the material and the ability to achieve high *R*_*solar*_ change. However, it has limitations, such as the need for enclosed structures for effective phase transition of the refractive index-matched liquid and additional external structures like fluidic channels and gas pumps for liquid/gas transport [12]. Also, there are still challenges to overcome regarding the high boiling point of the refractive index-matched liquid [15], which limits the practicality in real usage. The second thermal management strategy is using mechanical mechanisms that involve manipulating optical properties of surface by flipping, rotating, lifting, and stretching/compressing. These approaches are highly sustainable as they offer efficient switching with low energy requirements and outstanding regulation of radiative thermal performance by utilizing distinct radiative cooling and solar heating layers. Furthermore, their simplicity, which does not necessitate phase changes in liquids, allows for the straightforward construction of diverse system configurations with fast switching capabilities. However, it is important to note that these methods entail mechanical moving components within the system, which can pose challenges in terms of system installation, maintenance, and management. Third, the thermochromic materials whose optical properties vary with temperature caused by the phase transition in the crystal/molecular structure of the materials show radiative thermal management performance. Thanks to their self-adaptive modulation capabilities, radiative thermal management for *R*_*solar*_ or *ε*_*LWIR*_ is achievable without additional systems or components for switching. However, despite some degree of control over the transition temperature, there are limitations in adjusting *T*_*C*_ to adapt to dynamic climates, which can pose challenges in applying these methods across diverse climate environments [77–81]. Finally, the electrochromic mechanism leverages liquid crystals with alignment properties in response to an electric field or IR-reflective metal electrodeposition to control *R*_*solar*_ or *ε*_*LWIR*_ in thermal management devices. This approach offers advantages like low driving voltage, ultrafast switching speed [26], and notably high LWIR modulation performance. However, electrochromic device fabrication can be relatively complex, and it tends to focus primarily on *ε*_*LWIR*_ modulation, representing a limitation of this method [73, 82]. Table 1 summarizes a set of research categorized by the mechanisms mentioned above.

**Table 1:** Summary of radiative thermal management depending on mechanism, materials, spectral range, modulation and performance.

Ref.	Mechanism	Materials	Spectral range	Modulation	Performance
[11]	Wetting/drying	PVDF/CA hierarchical porous coating	Solar	*R*_*sola*r_ = 96.6 %, *ε*_*LWIR*_ > 96 % (cooling) *T*_*solar*_ = 86.6 % (heating)	Annual energy saved 199,260 kWh
[12]	Wetting/drying	PVDF/PTFE porous polymer coating	Solar	*ΔT*_*solar*_ = 68 %	Sub-ambient radiative cooling of 3.2 °C Above-ambient solar heating of 21.4 °C
[13]	Wetting/drying	PVDF porous structure	Solar	*R*_*solar*_ = 95.3 %, *ε*_*LWIR*_ = 93.4 % (cooling) *T*_*solar*_ = 70 % (heating)	Sub-ambient temperature drop of 3.7 °C at noon
[14]	Wetting/drying	Porous PTFE layers with a cermet-based absorber	Solar	*ΔT*_*sola*_ = 62 %	Annual cooling and heating savings of 77 % and 27 %, respectively
[15]	Wetting/drying	Porous SiO_2_ nanoparticle coating	Solar	*ΔT*_*solar*_ = 80 %	Temperature increase of 10 °C in cold weather Temperature reduction of 5 °C in hot weather
[16]	Rolling	Cu/Zn and Cu for heating, Ag and PDMS for cooling	Solar/LWIR	*R*_*solar*_ = 97.3 %, *ε*_*LWIR*_ = 94.1 % (cooling) *α*_*solar*_ = 93.4 %, *ε*_*LWIR*_ = 14.2 % (heating)	19.2 % heating and cooling energy saving (1.7 times higher than cooling-only and 2.2 times higher than heating-only methods)
[17]	Stretching	Nanoparticle-embedded PDMS thin film with grating structure	LWIR	*Δε*_*LWIR*_ = 64 %	*ΔT* = 20.86 °C (temperature difference between −10 % and 60 % of strain)
[18]	Flipping	Porous PVDF/MXene hierarchical structure	Solar/LWIR	*R*_*solar*_ = 96.7 %, *ε*_*LWIR*_ = 96.1 % (cooling) *α*_*solar*_ = 75.7 %, *ε*_*LWIR*_ = 11.6 % (heating)	Daytime sub-ambient cooling of 11.7 °C Above-ambient solar heating of 8.1 °C
[19]	Rotating	PVDF-phase change membrane integrated structure	Solar/LWIR	For cooling, *R*_*solar*_ = 92 %, *ε*_*LWIR*_ = 81 % For heating, *α*_*solar*_ = 90 %, *ε*_*LWIR*_ = 1 %	*ΔT* = 25 °C
[20]	Rolling	PMP/TiO_2_ nanoparticle composite for cooling, Cr nanoparticles for heating	Solar/LWIR	For cooling, *R*_*solar*_ = 85 %, *ε*_*LWIR*_ = 97 % For heating, *α*_*solar*_ = 91 %, *ε*_*LWIR*_ = 8 %	Heating power of 859.8 W m^−2^ Cooling power of 126.0 W m^−2^
[21]	Stretching	Porous PDMS/carbon black particle layer	Solar	For cooling, *R*_*solar*_ = 93 %, *ε*_*LWIR*_ = 94 % For heating, *α*_*solar*_ = 95 %	Sub-ambient cooling in hot weather = 5 °C Heating in cold weather = 18 °C
[22]	Thermochromic	Poly(N-isopro-polyacrylamide-co-N, N-dimethyl acrylamide) hydrogels	Solar	*ΔT*_*solar*_ = 88.84 %	Temperature drop of 7.3 °C in summer Temperature increase of 3 °C in winter
[23]	Thermochromic	PVDF@PNIPAm film	Solar	*ΔT*_*solar*_ = 86.3 %, *ΔR*_*solar*_ = 70.0 %	*ΔT* = 9 °C
[24]	Thermochromic	VO_2_-based thermochromic window	LWIR	*Δε*_*LWIR*_ = 58 %	–
[25]	Thermochromic	VO_2_-based thermochromic window	LWIR	*Δε*_*LWIR*_ = 40 %	Energy saving of 324.6 MJ m^−2^
[26]	Electrochromic	Polymer-dispersed E7 liquid crystal window	Solar	*ΔT*_*solar*_ = 56.5 %	*ΔT* = 7 °C
[27]	Electrochromic	Electrochromic device with Cu(ClO_4_)_2_ electrolytes	LWIR	For cooling, *α*_*solar*_ = 33 %, *ε*_*LWIR*_ = 94 % For heating, *α*_*solar*_ = 60 %, *ε*_*LWIR*_ = 20 %	–
[28]	Electrochromic	Electrochromic device with Cu(ClO_4_)_2_ electrolytes	LWIR	*Δε*_*LWIR*_ = 85 %	107.7 MBtu of HVAC energy consumption all year (2.23 times larger than it can save on the new construction buildings)
[29]	Electrochromic	Electrochromic device with AgNO_3_, CuCl_2_ electrolytes	LWIR	*ΔR*_*solar*_ = 72 %	23 % annual HVAC energy consumption of a medium office building

**Figure 7:**
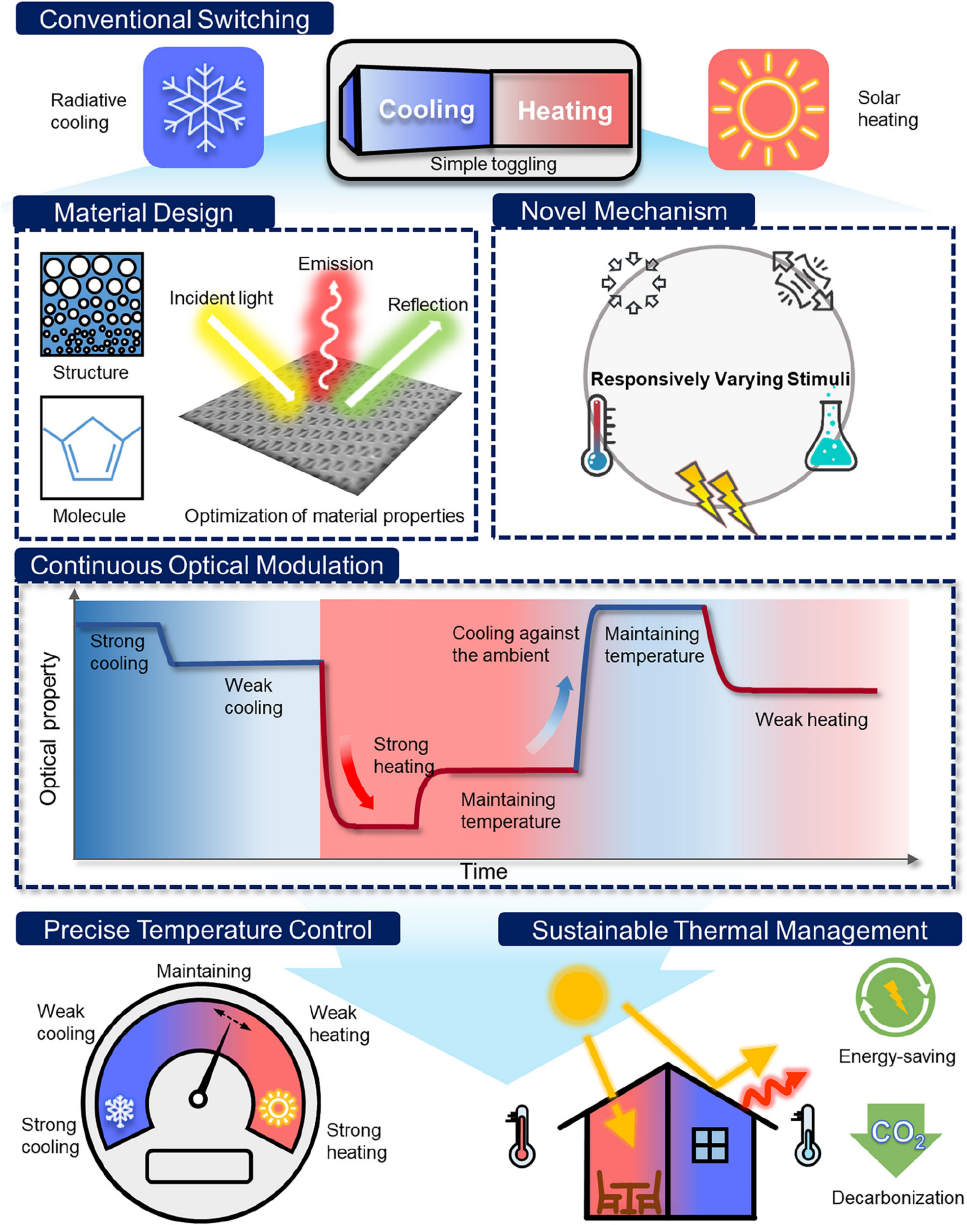
Conceptual illustrations of realizing advanced sustainable radiative thermal management. Reproduced with permission from Ref. [83] under the terms and conditions of the Creative Commons Attribution CC BY 4.0 license. Copyright 2023 Ma et al. published by Springer Nature.

Therefore, for radiative thermal management technologies to be further developed and be practically utilized for sustainable and effective thermoregulation, the following points should be considered (Figure 7): Firstly, research should focus on materials with exceptional optical and thermal properties while simultaneously developing a practical and new concept of high-performance modulating mechanisms. Secondly, it is crucial to focus on not just one parameter but on both *R*_*solar*_ and *ε*_*LWIR*_ for optical modulation performance. Solely controlling *R*_*solar*_ can lead to unwanted cooling during sunless nights or cloudy days, while solely controlling *ε*_*LWIR*_ cannot effectively manage the strong radiative power of solar irradiance. Therefore, achieving efficient radiative thermal management necessitates the control of both aspects. Lastly, the capability for precise and continuous radiation control should extend beyond simply toggling between radiative cooling and solar heating modes or merely switching radiative cooling on and off. Precise temperature control is important not only to effectively adapt to dynamic temperature changes [55] but also because each individual has a different preferred temperature for comfort. In addition, continuous optical modulation for temperature control is likely to have energy-efficient advantages compared to the repetitive switching between radiative cooling and solar heating modes to achieve the desired temperature. For instance, the PDLC film-based radiative cooling device developed by Deng et al. [26] showcases the potential for fine temperature control by continuously modulating *R*_*solar*_, ranging from low to high, through the control of the magnitude of the voltage. Also, Liu et al. [17] and Li et al. [55] proposed the concept of controlling *R*_*solar*_ and *ε*_*LWIR*_ continuously for precise modulation of cooling/heating temperature by adjusting mechanical strain. Furthermore, if *R*_*solar*_ and *ε*_*LWIR*_ can be precisely controlled over a broad range through switching mechanisms, it would be possible to achieve not only sub-ambient cooling and above-ambient heating but also continuously tracking temperature with specified target temperatures. This next-generation radiative thermal management combines the advantages of passive radiative cooling and solar heating, overcoming the limitations of conventional temperature control systems regarding carbon emissions. Simultaneously, it offers continuous thermal tunability, which has the potential to both practicality and reduce unnecessary cooling/heating energy consumption, making a significant contribution to global energy savings and decarbonization efforts. We expect that this perspective will offer valuable insights and exert significant influence on future research endeavors.
